# Amaryllidaceae Alkaloids Decrease the Proliferation, Invasion, and Secretion of Clinically Relevant Cytokines by Cultured Human Colon Cancer Cells

**DOI:** 10.3390/biom12091267

**Published:** 2022-09-09

**Authors:** Veronique Mathieu, Breana Laguera, Marco Masi, Sara Adriana Dulanto, Tanner W. Bingham, Lucas W. Hernandez, David Sarlah, Antonio Evidente, Denis L. J. Lafontaine, Alexander Kornienko, Michelle A. Lane

**Affiliations:** 1 Department of Pharmacotherapy and Pharmaceutics, Université libre de Bruxelles (ULB), 1050 Brussels, Belgium; 2 ULB Cancer Research Center, Université libre de Bruxelles (ULB), 1050 Brussels, Belgium; 3 Department of Chemistry and Biochemistry, Texas State University, San Marcos, TX 78666, USA; 4 Department of Chemical Sciences, University of Naples Federico II, 80126 Naples, Italy; 5 Roger Adams Laboratory, Department of Chemistry, University of Illinois, Urbana, IL 61801, USA; 6 Cancer Center at Illinois, University of Illinois, Urbana, IL 61801, USA; 7 RNA Molecular Biology, Fonds de la Recherche Scientifique (F.R.S/FNRS), Université libre de Bruxelles (ULB), Biopark Campus, 6040 Gosselies, Belgium; 8 Nutrition and Foods Program, School of Family and Consumer Sciences, Texas State University, San Marcos, TX 78666, USA

**Keywords:** colorectal cancer, Amaryllidaceae, narciclasine, pancratistatin, lycorine, haemanthamine, invasion, metastasis, MMP, VEGF, pentraxin 3

## Abstract

Alkaloids isolated from members of the *Amaryllidaceae* plant family are promising anticancer agents. The purpose of the current study was to determine if the isocarbostyrils narciclasine, pancratistatin, lycorane, lycorine, crinane, and haemanthamine inhibit phenomena related to cancer progression in vitro. To achieve this, we examined the proliferation, adhesion, and invasion of cultured human colon cancer cells via MTT assay and Matrigel-coated Boyden chambers. In addition, Luminex assays were used to quantify the secretion of matrix metalloproteinases (MMP) and cytokines associated with poor clinical outcomes. We found that all alkaloids decreased cell proliferation regardless of TP53 status, with narciclasine exhibiting the greatest potency. The effects on cell proliferation also appear to be specific to cancer cells. Narciclasine, lycorine, and haemanthamine decrease both adhesion and invasion but with various potencies depending on the cell line. In addition, narciclasine, lycorine, and haemanthamine decreased the secretion of MMP-1, -2, and -7, as well as the secretion of the cytokines pentraxin 3 and vascular endothelial growth factor. In conclusion, the present study shows that Amaryllidaceae alkaloids decrease phenomena and cytokines associated with colorectal cancer progression, supporting future investigations regarding their potential as multifaceted drug candidates.

## 1. Introduction

Colorectal cancer (CRC) is the third most common cause of cancer-related deaths in the United States. An estimated 151,030 new cases and 52,580 deaths are projected in the United States in 2022 [1]. Five-year survival rates depend on the cancer stage at the time of diagnosis. Localized disease, while found in less than 40% of primary diagnoses, has a 5-year survival rate of nearly 90%. Unfortunately, approximately 50% of all CRC patients will develop metastases, usually in the liver, in the course of their disease. When the disease has spread to regional lymph nodes, over 70% of patients will survive 5 years. In stage IV with distant metastases, found in more than 20% of primary diagnoses, the overall 5-year survival is 14% [1,2,3].

Progress toward improving the survival of metastatic CRC patients remains limited. Even with the best treatment after hepatic metastases have been detected the median survival is 6 months [2]. Surgical resection offers the best survival benefit, as chemotherapy or biologic agents provide only a marginal survival advantage when compared to supportive care alone. The current standard of care chemotherapeutic agents 5-fluorouracil and folinic acid (5-FU/LV) are effective in just 20% in CRC patients with hepatic metastases [3]. Lack of effectiveness is often due to p53-mediated chemoresistance [4] reflecting the loss of function mutations in TP53 found in approximately 70% of colorectal tumors [5]. TP53 loss of function mutations are linked not only to 5-FU resistance, but also to resistance to other chemotherapeutic drugs such as cisplatin [6,7]. When taken together, the high incidence of metastatic CRC combined with the lack of effective treatments for this disease, support the development of new chemotherapeutic agents that both shrink tumors and prevent metastatic disease.

**Figure 1:**
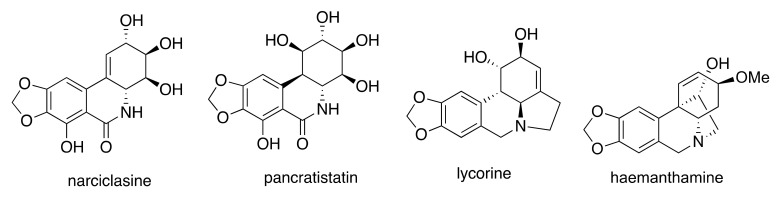
Molecular structures of the Amaryllidaceae alkaloids used in the present study.

Our laboratories have been pursuing the discovery of novel anticancer agents based on several alkaloids isolated from the Amaryllidaceae plant family [8,9,10,11,12,13,14]. The useful anticancer properties of a member of this plant family, *Narcissus poeticus* L., were known to Hippocrates of Cos (ca. B.C. 460–370), who treated uterine tumors with a pessary prepared from narcissus oil [15]. To date, more than 600 alkaloids of 15 chemical groups exhibiting various biological activities have been isolated from the Amaryllidaceae plants [16]. Most of these alkaloids belong to the lycorane and crinane chemical groups; however, the majority of the scientists investigating these plants as a source of anticancer agents share the opinion that the isocarbostyril non-basic constituents of the Amaryllidaceae, such as narciclasine (NAR) and pancrastitatin (PANC) (Figure 1), are likely to be the most important plant metabolites responsible for the anticancer properties of these plant species in folk medicine. Indeed, isocarbostyrils NAR and PANC are nanomolar antiproliferative agents in cancer cell cultures [16]. Notably, *Narcissus poeticus* L., used by Hippocrates, is now known to contain some 0.12 g of NAR per kg of fresh bulbs [17]. The NIH Developmental Therapeutics Database (DTD) 60 cell line screen predicts that the alkaloids LYC (LYC; lycorane group) and haemanthamine (HAE; crinane group) are less potent than NAR [18]. Despite these predictions, these compounds are of interest to our groups not only due to the dire need for new chemotherapeutic compounds to treat metastatic CRC but because the Amaryllidaceae alkaloids, in particular NAR and LYC, exhibit anti-inflammatory properties [19,20,21,22].

The link between inflammation and CRC progression is well known [23,24,25]. The four key pathways linking inflammation and cancer progression are nuclear factor kappa B (NF-κB), Janus kinase (JAK)-signal transducer and activator of transcription (STAT), mitogen activated protein kinase (MAPK) and phosphatidylinositol 3-kinase (PI3K)/Akt (protein kinase B) [26]. Of note, loss of p53 function is associated with increased tumor incidence and size in the presence of inflammation [27]. Loss of p53 function has also been linked to increased NF-κB signaling, the epithelial mesenchymal transition (EMT), and cell invasion [26]. LYC and NAR counteract lipopolysaccharide-induced increases in interleukin (IL)-1β, IL-6, and tumor necrosis factor alpha (TNF-α) levels via the inhibition of the NF-κB, c-Jun N-terminal kinases (JNK), and/or signal transducer and activator of transcription 3 (STAT3) pathways. This occurs in various in vitro and in vivo models of osteoarthritis, acute organ injuries and fibrosis, sepsis, and myocardial dysfunction [20,28,29,30,31,32,33,34,35,36].

To date, only few studies have evaluated the potential of Amaryllidaceae alkaloids to combat CRC. Specifically, LYC has been investigated in two studies highlighting its antiproliferative, pro-apoptotic, anti-migratory and anti-invasive properties both in vitro and in vivo [37,38]. PANC was shown to trigger apoptosis via the mitochondrial pathway independent of p53 status [39]. A PANC analog shared similar effects against cultured human colon cancer cells [40] and a PANC salt provoked tumor necrosis associated with vascular shutdown [41].

In the current study, we began by investigating the ability of the Amaryllidaceae alkaloids NAR, PANC, LYC, and HAE to decrease the proliferation of a panel of colon cancer cell lines with varying p53 functionality. The compounds chosen represent different subgroups of the most potent and abundant alkaloid groups in the Amaryllidaceae plant family (Figure 1). We then focused our work to examine the effects of NAR, LYC, and HAE on processes and proteins associated with CRC progression, specifically cell adhesion and invasion, matrix metalloproteinases (MMPs), and cytokines.

## 2. Materials and Methods

### 2.1. Cell Lines and Cultures

HCT-116 (CCL-247), LoVo (CCL-229), DLD-1 (CCL-221), HT-29 (HTB-38) and CoN (CCD-841) human CRC cells were obtained from the ATCC (Manassas, VA, USA). HCT-116 cells were cultured in Dulbecco’s Modified Eagle’s Medium (DMEM; Corning Cellgro, Corning, NY, USA) for the invasion assays or RPMI-1640 medium (Lonza, Westburg, The Netherlands) for all other assays. The LoVo, DLD-1, and HT-29 cell lines were grown in RPMI-1640 for all experiments. Media were supplemented with 10% fetal bovine serum (FBS; Gibco, Waltham, MA, USA or Greiner Bio-One, Vilvoorde, Belgium), 100 U/mL penicillin, and 100 µg/mL streptomycin (Gibco, Waltham, MA). Cells were grown at 37 °C and 5% CO_2_ in a humidified atmosphere. The CoN cells were cultured in Eagle’s Minimum Essential Medium (Lonza, Westburg, The Netherlands) supplemented with 10% FBS (South America origin, Greiner Bio-One, Vilvoorde, Belgium). HCT-116 p53^-/-^ cells were obtained from Denis L.J. LaFontaine, Université Libre de Bruxelles, Brussels, Belgium [42].

### 2.2. Compounds

LYC was obtained from dried bulbs of *Sterbergia lutea* Ker Gawl by acid extraction as reported in [43]. Briefly the crude alkaloid obtained by alkalinization of the plant acid extract was crystallized as white prisms from ethanol obtaining 11.2 g starting from 2.5 kg of dried bulbs (4.5 g/kg). NAR and HAE were obtained by Soxhlet extraction with ethanol from dried bulbs (6 kg) of *Narcissus pseudonarcissus* King Alfred. The organic extract (4.5 g) was purified by flash/chromatography on SiO_2_ eluted with ethyl acetate:methanol:H_2_O (85:10:5) yielding 10 homogeneous fractions (F1–F10). The residue of F3 consisted of pure NAR (166 mg/kg), while that of F6 similarly gave pure HAE (465 mg/kg). The chromatographic and spectral characteristics of LYC, NAR and HAE were identical to the samples available in our laboratory, whose data were published previously [9,13,14]. Pancratistatin was obtained by total synthesis [44].

### 2.3. Viability Assay

Effects of compounds on viability were assessed by means of 3-(4,5-dimethylthiazol-2-yl)-2,5-diphenyltetrazolium bromide (MTT; Sigma-Aldrich, Diegem, Belgium) colorimetric assay as previously reported [45]. Briefly, cells were seeded in flat-bottom 96-well plates at the appropriate cell density 24 h prior to the exposure or not to the compound of interest. Each compound was first solubilized in dimethylsulfoxide (DMSO) at 10^−2^ M concentration and further diluted in a serial manner in the culture medium. Nine concentrations ranging from 10 nM to 100 µM were tested in sextuplicate within the assay. Evaluation of the cell viability was conducted after 72 h of exposure to the compound by means of MTT assay [46].

### 2.4. Adhesion Assay

To evaluate the effects of the compounds on cell adhesion, 75000 HCT-116 or LoVo cells per well were seeded in 96-well flat-bottom plates in the presence or absence of each compound tested at three concentrations. In the first set of experiments, cells were allowed to adhere for 1, 5, 8, and 24 h and cultured with 10% FBS. Three independent experiments were performed each in sextuplicate to examine the time course of adhesion when grown with 10% FBS and increasing concentrations of each compound. In the second set of experiments cells were allowed to adhere for 15 h in a medium containing only 0.5% FBS to limit the contribution of proliferation to the results.

At the end of the incubation period, the plate was rinsed with phosphate buffered saline (PBS), fixed with 17.5% of formaldehyde at 4 °C for 15 min, and washed with deionized water twice before staining with 0.1% crystal violet (Sigma-Aldrich, Diegem, Belgium) in a 50:50 methanol/water mixture for 15 min. After extensive washes with water to remove any free crystal violet, the dye within the cells was extracted and solubilized in 100 µL of a 33% acetic acid solution and absorbance was measured at 570 nm (680XR, Bio-Rad Laboratories, Berkeley, CA, USA; reference wavelength 610 nm). The mean absorbance of the untreated control well was set at 100% for comparison. For the second set of experiments, each condition was seeded in parallel in two plates. The duplicate plate was used to evaluate the viability of the cells. Those were first centrifuged for 8 min before removing the supernatant to keep the non-adherent cells for viability assessment through MTT assay as described above in Section 2.3.

### 2.5. Invasion Assay

Prior to seeding HCT-116 cells in the invasion chambers, the medium was removed and cells were washed twice with phosphate buffered saline and incubated in DMEM lacking FBS for 48 h before seeding at a density of 1 × 10^5^ cells/well into the upper portion of Matrigel-coated invasion chambers (Corning Biocoat, Corning, NY, USA), as described previously by our group in [47,48]. The upper portion of each chamber contained cells suspended in serum-free DMEM containing DMSO vehicle alone or increasing concentrations of the Amaryllidaceae alkaloids NAR, LYC, and HAE. In each case, the volume of DMSO (Sigma-Aldrich, St. Louis MO) vehicle did not exceed 0.1% and all wells received the same volume of DMSO. The lower portion of each chamber contained DMEM supplemented with 10% FBS. The FBS served as a chemoattractant. A polyethylene terephthalate (PET) membrane, containing 8 μm pores and coated with Matrigel basement membrane matrix, separated the upper and lower portions of the invasion chamber. After 24 h, chambers were removed and the cells that had not invaded were removed from within the chamber using a cotton swab. The cells that had invaded through and adhered to the bottom of the PET membrane separating the chambers were fixed with methanol and stained with 0.5% crystal violet (Sigma-Aldrich, St. Louis, MO, USA). Five random images were taken of the bottom of each membrane using a Cytation 5 Cell Imaging Multi-Mode Reader (Winooski, VT, USA). The cells in each image were counted using Image J (NIH, Bethesda, MD, USA).

To correct for the inhibitory effects of the Amaryllidaceae alkaloids on cell proliferation, cells from the same biological repeat were seeded in parallel in 24-well tissue culture plates at a density of 1 × 10^5^ cells/well in DMEM containing 5% FBS (the average of the FBS concentrations in the upper and lower chambers) and supplemented with vehicle or increasing concentrations of the alkaloids. These wells were not coated with Matrigel. The medium was removed, and the adherent cells were trypsinized and counted 24 h after treatment using a hemocytometer. To compare cell invasion across treatments, cell invasion was corrected for adherent cell proliferation and expressed as % vehicle control. 

Experiments with LoVo cells were carried out similarly but the culture medium was RPMI-1640 and cell seeding was 80,000 cells per well. In addition, due to supply problems, the coating of the invasion chambers was performed manually with 100 µL of a Matrigel solution at 200 µg/mL made in PBS allowed to polymerize for 2 h at 37 °C as recommended by the manufacturer. The staining of the invasive cells was performed with Hemacolor (Sigma-Aldrich) and the counting of the wells seeded in parallel was performed with a Countess II apparatus (ThermoFisher Scientific, Merelbeke, Belgium).

### 2.6. Luminex Assay

We ordered a customized Luminex^®^ assay to quantify 15 targets at once from Biotechne (reference LXSAHM-15; R&D Systems, Abingdon, U.K.). The 15 targets were chosen on the basis of the literature as follows: the relevant metalloproteinases MMP-1, -2, -7, -9, -13 [49] and the cytokines associated with dismal prognosis or shorter disease-free survival period: C-C motif chemokine ligand (CCL) -4, CCL-22, Flt-3 ligand, IL-1β, IL-2, IL-6, IL-8, interferon gamma (IFNγ), vascular endothelial growth factor (VEGF), and pentraxin-3 [50]. Samples were prepared as follows: HCT-116 or LoVo cells were seeded in 6-well plates and allowed to adhere and grow until they reached 70% confluency. The culture medium was replaced by 1.5 mL of culture medium containing only 0.5% of FBS with or without the compounds to be tested (NAR at 80 nM, LYC at 6 µM, and HAE at 6 µM). After 24 h of treatment, supernatants were collected, aliquoted, and kept frozen at −80 °C. Three independent series of samples were prepared. A preliminary assay conducted on one sample of each condition revealed that the signal was too low for several targets. We thus decided to concentrate several aliquots of our supernatant samples by centrifugation of 500 µL of supernatant with Amicon Ultra 0.5 mL filters characterized by a 3 kDa cut-off (UFC500324; Millipore, Merck, Hoeilaart, Belgium) leading to a final volume of 150 µL (3.3 times concentrated). The Luminex^®^ assay was conducted according to manufacturer’s recommendations. Data were collected on a Bioplex-200 (Bio-Rad, Temse, Belgium).

### 2.7. Statistical Analyses

All data are expressed as the mean ± standard error of the mean (SEM) of three separate experiments unless otherwise noted in the figure itself or its legend. Significant effects were determined using Mann–Whitney U-tests performed in SPSS v24 (IBM, Armonk, NY, USA) or Statistica v7.0 comparing each concentration of alkaloid to control. Differences were considered significant at *p* < 0.05.

## 3. Results

### 3.1. Antiproliferative Effects of Amaryllidaceae Alkaloids in Colon Cancer Cell Lines

We determined the growth inhibitory potential of the Amaryllidaceae alkaloids NAR, PANC, LYC, and HAE against five human colon cancer and cell lines and one non-cancerous, colon epithelial cell line. Table 1 shows that NAR and PANC appeared the most potent Amaryllidaceae alkaloids with IC_50s_ in the nanomolar range. In this assay, NAR was 10 times more potent than its closed analog, PANC. HAE and LYC harbored similar potencies against all cancer cell lines, regardless of TP53 status, with IC_50s_ close to 3 µM after 72 h of exposure to the compounds. Our data for NAR, PANC, and LYC are consistent with the NCI DTD’s 60 cell line screen database. In this screen against the subpanel of CRC cell lines NAR, PANC, and LYC demonstrated mean GI_50s_ of 45.7 nM, 262.8 nM, and 1.99 µM, respectively [18]. HAE, on the other hand, performed better against our cells than against the NCI subpanel of the CRC cell lines where it registered a mean GI_50_ of 15.8 µM.

Because TP53 mutations are common in colon cancer patients [5], cisplatin, a drug whose activity relies on functional p53, was included for comparison [6,7]. In addition, the five cell lines used differ in TP53 status. Specifically, the LoVo cell line expresses wild type, functional p53 as does the HCT-116^+/+^ cell line, while the corresponding HCT-116^-/-^ line is p53-null. The HT-29 and DLD-1 cell lines harbor different single mutations in TP53, reducing its function [51,52]. Importantly, the Amaryllidaceae alkaloids were effective in TP53 null or mutant cell lines. For example, NAR, PANC, and HAE displayed similar effects toward both HCT-116^+/+^ and HCT-116^-/-^ models while cisplatin was about four times less active against the HCT-116^-/-^ cells, as expected. Similarly, cisplatin was also less potent against the HT-29 and DLD-1 p53-mutant cell lines (Table 1; [7]). LYC appeared two times more potent against HCT-116^+/+^ than HCT-116^-/-^ models. However, considering the comparison between the IC_50s_ obtained with the LoVo cell line (TP53 wild type) and the mutant cell lines (HT-29 and DLD-1), it remains unclear whether the difference between the HCT-116 models observed with LYC is biologically significant and related to p53 functionality.

Testing also included one non-cancerous normal human epithelial colon cell line, i.e., the CoN cell line. Comparison of the mean IC_50s_ of cancer cell lines with their cytotoxic activity against the CoN cell line revealed that NAR, LYC, and HAE were about 25 to 30 times less toxic toward non-cancerous cells. For comparison, cisplatin was only 5 times less toxic. These data suggest that these Amaryllidaceae alkaloids are more potent than a current chemotherapeutic agent, they are effective regardless of TP53 status, and their antiproliferative effects target cancerous cells in preference to normal ones. Considering that PANC is less active and possibly less selective than its very closed analog NAR, we focused our study on NAR, LYC, and HAE, the three most potent Amaryllidaceae alkaloids representative of the different isocarbostyril, lycorane, and crinane chemical subfamilies.

### 3.2. In Vitro Evaluation of Anti-Invasive Effects of Amaryllidaceae Alkaloids

**Figure 2:**
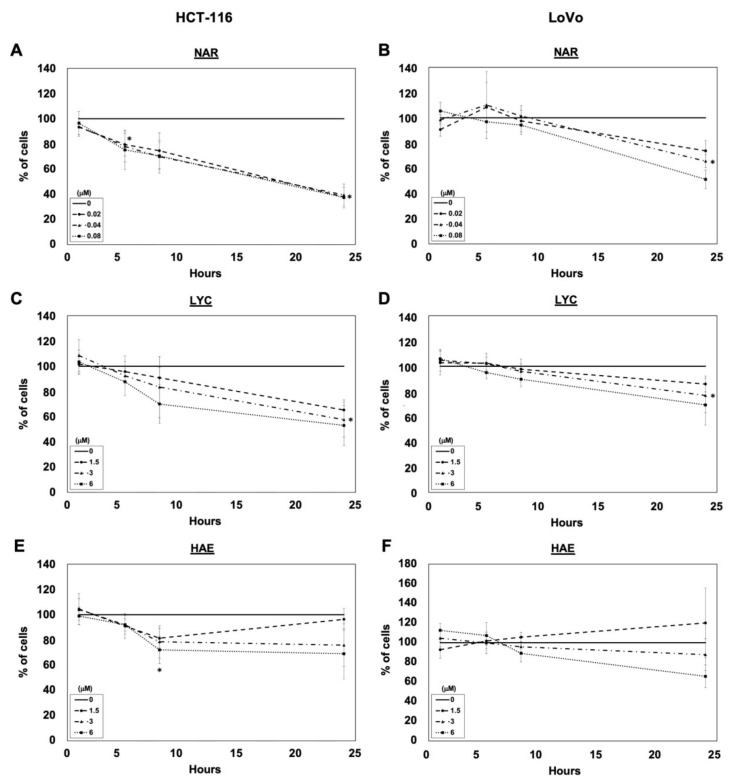
The anti-adhesive effects of increasing concentrations of Amaryllidaceae alkaloids over time. The relative proportion of adherent HCT-116 (left column) and LoVo (right column) cells 1, 5, 8, and 24 h after seeding in the presence of 10% FBS and (**A**,**B**) narciclasine (NAR), (**C**,**D**) lycorine (LYC), and (**E**,**F**) haemanthamine (HAE) versus untreated cells (control, 0). Solid lines indicate control, dashed lines half IC_50_, dashed and dotted lines IC_50_, and dotted lines twice IC_50_. Adherent cells were stained with crystal violet. The absorbance of untreated cells was normalized to 100%. Data are expressed as mean ± SEM of 3 independent experiments, plated in sextuplicate. * *p* < 0.05 vs. control for all three concentrations of Amaryllidaceae alkaloids at the specified time point.

Because members of our group use HCT-116 cell line to examine the effect of diet-derived compounds on cell invasion [47,48] and previous work examined the ability of LYC to decrease the invasion of the HCT-116 cell line (wild type) and the LoVo models [38], we focused on these models. We first evaluated whether treatment for 1, 5, 8 and 24 h with HAE, LYC, or NAR affected cell adhesion at three concentrations: one close to their mean IC_50_, one two times higher, and one two times lower in cells cultured with 10% FBS (Figure 2). At this FBS concentration, all three compounds inhibited cell adhesion in a dose-dependent manner. Cell adhesion was less affected by all these compounds in the LoVo cell line and HAE appeared to have the weakest effect in both cell lines. The inhibitory effects were greatest after 24 h of treatment. At that time point, possible contribution of antiproliferative effects cannot be ruled out.

**Figure 3:**
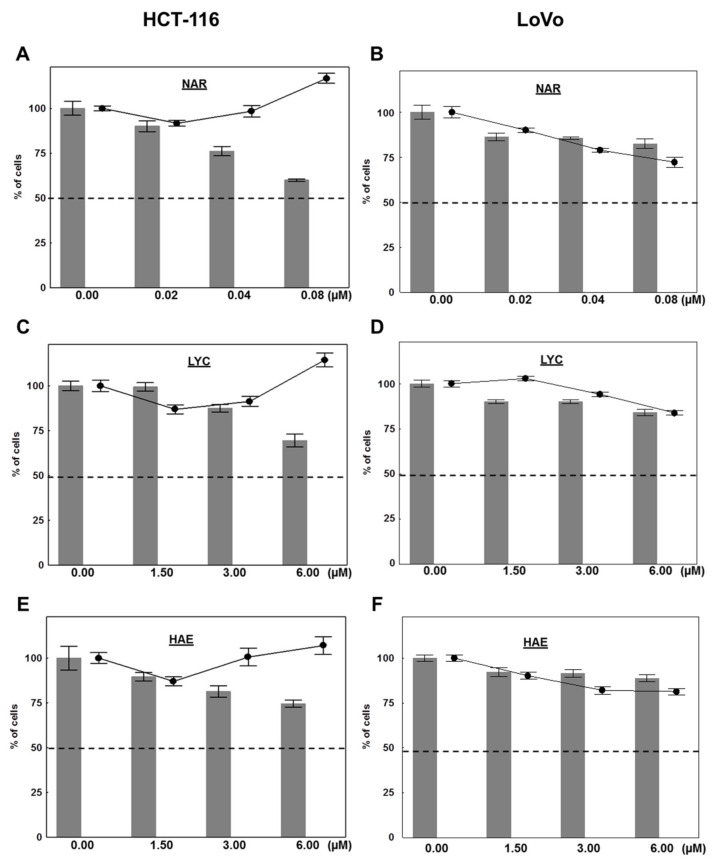
The anti-adhesive effects of Amaryllidaceae alkaloids in 0.5% FBS. Gray bars represent the relative proportion of adherent HCT-116 cells (left panels) or LoVo cells (right panels) 15 h after seeding in the presence of (**A**,**B**) narciclasine (NAR), (**C**,**D**) lycorine (LYC), and (**E**,**F**) haemanthamine (HAE) versus untreated cells (control, 0). Adherent cells were stained with crystal violet. The absorbance of untreated cells after the 15 h was normalized to 100%. Black circles represent the combined viability of both adherent and non-adherent cells assessed by a MTT assay performed in parallel with the adhesion experiment corresponding to the treatment indicated by the gray bar immediately to their left. Cells were centrifuged before MTT exposure and staining to capture non-adherent cells. Data are expressed as mean ± SEM of six replicates within one experiment. Again, the absorbance of untreated cells after the 15 h was normalized to 100%.

We thus performed a second set of experiments with cells cultured in 0.5% FBS, to decrease the effects of cell proliferation on adhesion. Adhesion was examined at 15 h to limit the contribution of proliferation in the results presented above. Figure 3 highlights that all three compounds impaired HCT-116 and LoVo adhesion in a dose-dependent manner 15 h after plating in the presence or absence of these compounds. Although the experiment was only conducted once, the results were highly significant regarding HCT-116 cells. According to results of Figure 2, LoVo cell adhesion was lesser impaired than the one of HCT-116 cells with all three compounds. Of note, the greatest inhibition of cell adhesion in HCT-116 cell line was observed with NAR at 0.08 µM (39%) while LYC and HAE appeared less potent at both their IC_50_ concentration (3 µM) and double that concentration (6 µM; Figure 3). Cell viability of both HCT-116 adherent and non-adherent cells evaluated in parallel (black circles) was not decreased below 87% in any treatment. By contrast, impairment of cell adhesion (maximal inhibition of 18% obtained again with NAR 0.08 µM) seems to be associated with reduced viability in LoVo cells suggesting that the latter harbor poorer resistance to anoïkis than HCT-116 cells (Figure 3). In conclusion, although the anti-adhesive properties of Amaryllidaceae alkaloids vary from one cell line to another, narciclasine appeared to be the most potent in both cases.

**Figure 4:**
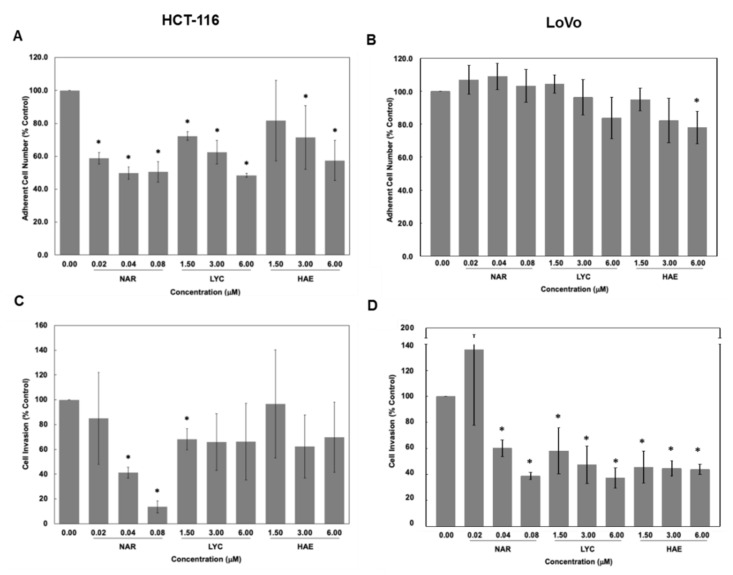
Effect of Amaryllidaceae alkaloids on HCT-116 and LoVo cell invasion. HCT-116 or LoVo human colon cancer cells were incubated with increasing concentrations of narciclasine (NAR), lycorine (LYC), and haemanthamine (HAE) and invasion through Matrigel-coated Boyden chambers was assessed as described in Materials and Methods. Five random fields of view were quantified per experiment. The effect of Amaryllidaceae alkaloids on adherent cell number was determined in parallel and expressed relative to vehicle control for HCT-116 (**A**) and LoVo cells (**B**) Cell invasion (**C**) HCT-116 cell line; (**D**) LoVo cell line was corrected for the effects of the Amaryllidaceae alkaloids on the number of adherent cells and expressed relative to vehicle control. Data are expressed as mean ± SEM, n = 3. * Significantly different that vehicle control, *p* < 0.05.

We next evaluated the impact of Amaryllidaceae alkaloids on cell invasion by means of Matrigel-coated Boyden chambers (Figure 4). Importantly, considering the lack of adhesion of cells in presence of the compounds as described above, we corrected the invasion rates to reflect the number of actual adherent cells measured in wells seeded in parallel. Correcting cell invasion for adherent cells allowed us to isolate the effects of these compounds on invasion, exclusive of their impact on cell viability, proliferation, and adhesion [47,48]. Figure 4A,B illustrate the percentage of adherent HCT-116 and LoVo cells 24 h after their seeding in parallel to the Boyden chambers, respectively. Reflecting the results of the adhesion assay in Figure 3A, we observed a dose-dependent decrease in adherent cells with all compounds but in a more marked manner with NAR (Figure 4A) in HCT-116 cells while the decrease was more limited in the LoVo cells (significant only with HAE 6 µM; Figure 4B). When corrected for adherent cells, the invasion data (Figure 4C,D) show that NAR strongly inhibited invasion independent of its effects on adhesive cell number. Specifically, treatment of HCT-116 cells with 0.04 and 0.08 µM NAR reduced cell invasion to 41.1 ± 4.3% and 13.6 ± 4.8% of vehicle control (Figure 4C). The dose-depending anti-invasive effects of NAR were also observed with respect to LoVo cells on which LYC and HAE also exerted potent effects (38.8 ± 2.8%, 37.4 ± 7.8% and 38.8 ± 2.8% of residual invasion rates with NAR 0.08 µM, LYC 6 µM and HAE 6 µM, respectively; Figure 4D). Because invasion through Matrigel-coated Boyden chambers requires that cells not only move through the membrane separating the well from the lower chamber but digest also Matrigel, a basement membrane-like mixture covering this membrane, we next examined the effect of Amaryllidaceae alkaloids on MMP secretion.

**Figure 5:**
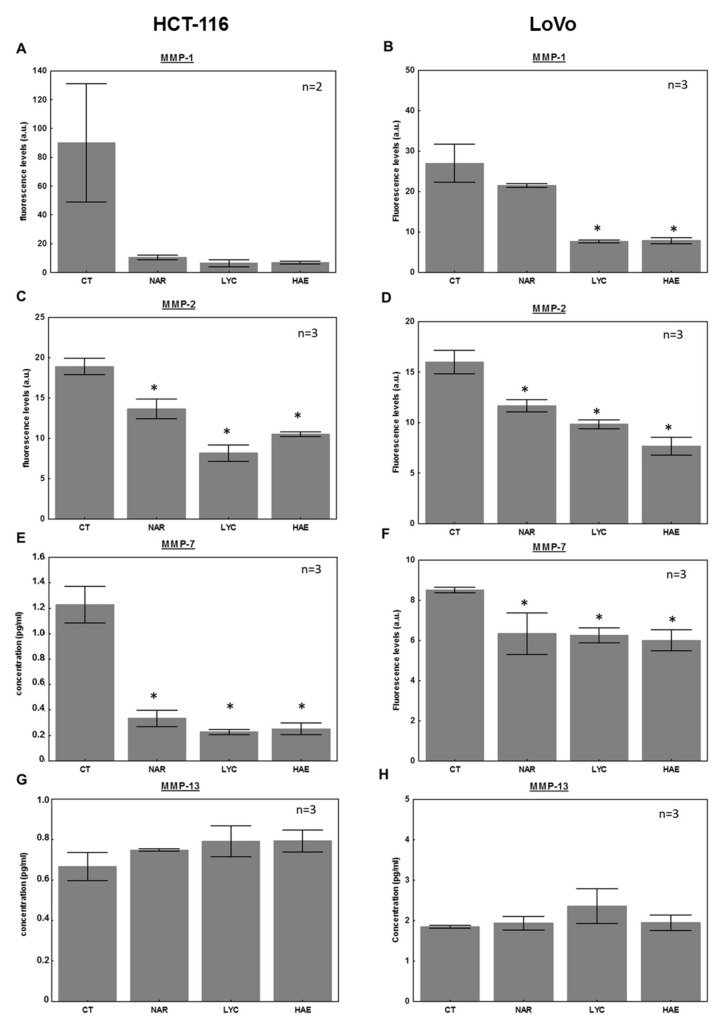
Effects of Amaryllidaceae alkaloids on matrix metalloproteinase (MMP) secretion. MMP levels in HCT-116 cell supernatants (left panel) and LoVo (right panel) cell supernatants were determined using a customized Luminex assay (see Materials and Methods). Cells were treated for 24 h with or without (control, CT) 0.8 µM narciclasine (NAR), 6 µM lycorine (LYC), or 6 µM haemanthamine (HAE). Data are expressed as mean ± SEM, n = 3 with the exception of (**A**) where n = 2. * Different from vehicle control, *p* < 0.05 (**B**–**H**).

For this purpose, we quantified the secreted levels of various MMPs shown to be associated with colon cancer metastases and prognosis in patients. MMP-1, -2, -7, -9 and -13 are markers of poor colon cancer prognosis [49]. Each of these proteins digests a component of the extracellular matrix, facilitating the intravasation and extravasation of tumor cells [53,54]. Specifically, MMPs 1 and 13 are collagenase, MMP-2 is a gelatinase, and MMP-7 digests a wide variety of proteins including collagens, gelatins, laminin, proteoglycans, fibronectin, and elastin. MMP-1 also induces PI3K/Akt signaling, associated with the EMT and metastasis [55]. We measured the levels of these MMPs in the supernatants of HCT-116 and LoVo cells treated at the highest concentration of each Amaryllidaceae alkaloid for 24 h, the same treatment duration as the invasion assay. NAR (0.08 µM), LYC (6 µM) and HAE (6 µM) appeared to markedly decrease the secretion of MMP-1, -2, and -7 in both cell lines (Figure 5A–F). No effect on MMP-13 was observed although both cell lines do secrete this MMP (see control condition, Figure 5G,H). Regarding MMP-7, a significant but lesser effect was observed, in particular in LoVo cell line (Figure 5E,F). No MMP-9 could be detected in the culture medium in any condition (levels below 0.04 pg/mL) using our Luminex assay although MMP-9 has been previously detected by other methods in HCT-116 [47,56] and LoVo [57] cell supernatants. Our results show for the first time that not only LYC but also NAR and HAE markedly decrease the levels of MMP-1, -2 and -7 all of which are associated with worse outcomes for CRC patients [49].

### 3.3. The Effect of Amaryllidaceae Alkaloids on Clinically Relevant Cytokines

**Figure 6:**
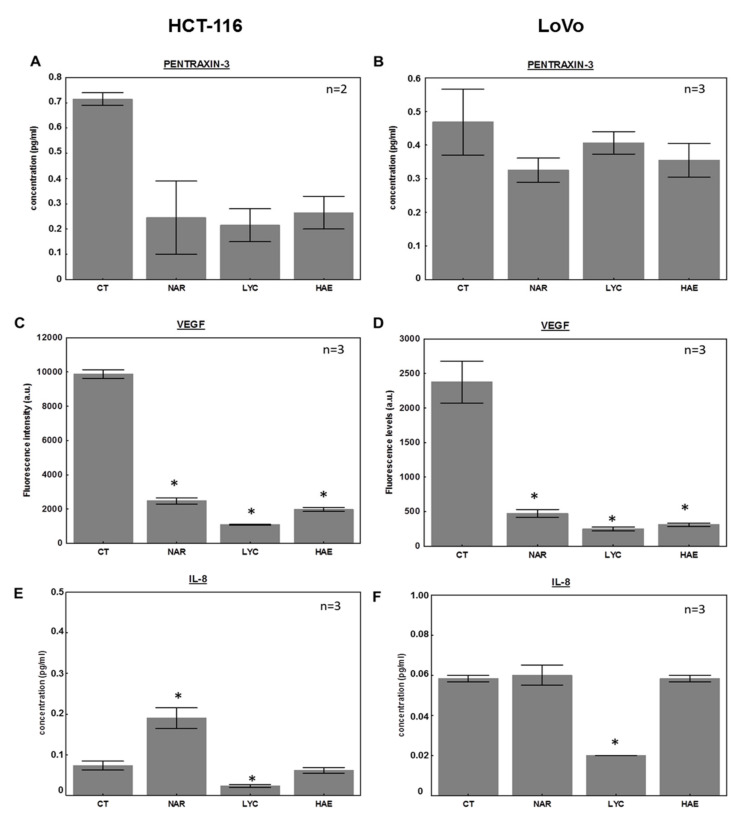
Effects of Amaryllidaceae alkaloids on cytokines involved in colon cancer progression. Levels of the cytokines pentraxin-3 (**A**,**B**), vascular endothelial growth factor (VEFG; **C**,**D**), and interleukin-8 (IL-8; **E**,**F**) in HCT-116 (left panel) and LoVo (right panel) cell supernatants were determined using a customized Luminex assay (see Materials and Methods). Cells were treated for 24 h with or without (control, CT) 0.8 µM narciclasine (NAR), 6 µM lycorine (LYC), or 6 µM haemanthamine (HAE). Data are expressed as mean ± SEM, n = 3 with the exception of (**A**) where n = 2. * Different from vehicle control, *p* < 0.05 (**C**–**F**).

Based on the recent review by Gunawardene et al. [50], we examined the effects of Amaryllidaceae alkaloids on cytokine levels found to be predictors of overall or disease-free survival for CRC patients; specifically IL-2, IL-6, IL-8, CCL-4, CCL-22, Flt-3 ligand, IFNγ, VEGF, and pentraxin 3. Importantly, IL-8 and IL-6 play crucial roles in promoting CRC migration and metastatic processes in addition to the tumor microenvironment and inflammation [58,59,60]. Unfortunately, the sensitivity of the assay allowed us to detect only IL-8, VEGF, and pentraxin 3 in our cell culture supernatants. Data suggest that all Amaryllidaceae alkaloids tested decrease pentraxin 3 and VEGF, proteins associated with poor prognosis of CRC patients (Figure 6A–D) [50]. Pentraxin 3 levels were decreased by two to three times depending on the compound (Figure 6A) in HCT-116 cells only. VEGF levels were decreased up to 5 times by NAR (0.08 µM) and to 10 times by LYC (6 µM) in both cell lines (Figure 6C–D). On the contrary, we observed opposite effects of NAR in comparison to LYC on IL-8 levels in HCT-116 cells (Figure 6E). As only the inhibitory effects of LYC on IL-8 were reproduced in both cell types, the effects of NAR on HCT-116 warrant further investigation. When taken together, these data indicate that the Amaryllidaceae alkaloids alter the secretion of cytokines in a manner associated with increased overall survival for CRC patients.

## 4. Discussion

Metastatic CRC has a poor survival rate and current chemotherapeutic regimens are often ineffective due to p53-mediated chemoresistance [2,3]. In the current study we first evaluated the antiproliferative effects of representative compounds of the main chemical groups of Amaryllidaceae plants including the most potent ones, NAR and PANC. We showed that all four compounds tested, i.e., NAR, PANC, LYC, and HAE exert potent antiproliferative effects on various human colon cancer cell lines harboring different p53 status with IC_50s_ ranging from 23 nM to 5.5 µM (Table 1). In each case, the Amaryllidaceae alkaloid exhibited greater potency than cisplatin. Because prevention of metastasis is key to increasing patient survival, we next determined the effect of Amaryllidaceae alkaloids in cell adhesion, cell invasion, and the secretion of MMPs associated with poor patient prognosis [49]. We focused on the three compounds that appeared to selectively target cancer cells, sparing normal cells, specifically, NAR, LYC, and HAE. Each of these alkaloids decreased cell adhesion (Figure 2 and Figure 3), but only NAR decreased cell invasion in both models used. All compounds also decreased the secretion of MMPs 1, 2, and 7 while none affected MMP-13 secretion. Finally, we examined the ability of NAR, LYC, and HAE to decrease the secretion of cytokines associated with poor patient prognosis. We found that all three alkaloids reduced strongly VEGF secretion. LYC reduced IL-8 secretion in both cellular models. Taken together, our results indicate that the Amaryllidaceae alkaloids NAR, LYC, and HAE hold potential for preventing CRC metastasis and increasing patient survival.

Overcoming p53 deficiency and its associated chemoresistance is one of the main challenges in combating CRC. Indeed, p53 functionality is altered in 70% of CRC [5]. In general, tumors are treated with 5-FU in combination with oxaliplatin and/or irinotecan as the first line of chemotherapy (FOLFOX, FOLFIRI, FOLFOXIRI; [61]). Still, p53 deficiency is associated with 5-FU and cisplatin resistance [6,7], while its deficiency seems to have less impact on irinotecan and oxaliplatin sensitivity as these compounds do not trigger mismatch repair proteins [4,7].

Amaryllidaceae alkaloids have been reported to cause both p53-dependent and -independent apoptosis in various cell lines, exclusive of colon cancer. For example, LYC was previously reported to trigger apoptosis in a p53-dependent manner in other cancer cell types such as osteosarcoma [62] and leukemia [63] but was not investigated, at least to the best of our knowledge, in p53-deficient models. By contrast, PANC and one of its derivatives were both reported to induce mitochondria-mediated apoptosis in HT-29 p53-deficient and HCT-116 p53 wild-type models [39,40]. It should be noted that these results were obtained at 1 µM PANC (while they observed an IC_50_ of 100 nM), suggesting that this cytotoxic effect does not depend on p53 [39]. HAE was also shown to induce dose-dependent apoptosis in a p53-null leukemia model [64].

We report here for the first-time similar effects for NAR, LYC, and HAE in cultured human colon cancer cell lines. The absence of influence of p53 on the antiproliferative effects of these Amaryllidaceae alkaloids might be surprising when considering that NAR, LYC, and HAE can bind not only to the mature ribosome [11,65] and consequently inhibit translation but also affect pre-rRNA precursors associated with the variable nucleolar stress response leading to stabilization of p53 [11]. This stabilization has been shown to be much more pronounced with HAE than LYC or NAR at equipotent concentrations in HCT-116 wild-type cells [11]. This suggests that other important effects independent of p53 may actually contribute to the ability of these compounds to reduce colon cancer cell proliferation in p53-deficient models and counteract p53-mediated resistance, as observed here.

Our invasion results (Figure 4) suggest that NAR exerts stronger anti-invasive effects than LYC and HAE, but this finding cannot be attributed to its influence on MMP-1, -2, -7, and 13 in the cell supernatant as all four alkaloids displayed similar effects on these MMPs (Figure 5). Treatment with 1.5 µM LYC also reduced the invasion of HCT-116 cells (Figure 4), but this effect was not observed in a dose-dependent manner in contrast to results obtained by Zhang et al. [37]. One explanation for this discrepancy may be that the authors of [37] did not take into account the dose-dependent effects on cell adhesion as observed in the present study with respect to HCT-116 (Figure 3). Indeed, we observed, similarly to Zhang et al. [37], dose-dependent effects of LYC on LoVo cells that are much less sensitive to its anti-adhesive effects (Figure 2, Figure 3 and Figure 4). In addition, our invasion data have been normalized to account for the effects on cell adhesion, but NAR may be a more potent inhibitor of HCT-116 cell motility. Indeed, we have previously shown that NAR, as well as LYC, exerts marked anti-migratory effects in glioma and melanoma cells depending on Rho A/ Rho-associated kinase (ROCK) and/or eukaryotic elongation factor 1 alpha (eEF1α)-mediated regulation of actin cytoskeleton polymerization and bundling [66,67,68]. Adhesion complexes are in close connection to the actin cytoskeleton. The more potent effects observed on cell adhesion with NAR (Figure 3) support our hypothesis that NAR may exert a greater impact on actin cytoskeleton dynamics and related phenomena, such as adhesion and motility, than the other Amaryllidaceae alkaloid chemical subgroups represented herein by LYC and HAE. This might be attributed to better inhibition of eEF1α, another direct target of Amaryllidaceae alkaloids, as demonstrated for NAR [68].

In the current study, we also observed that Amaryllidaceae alkaloids provoked a strong decrease in MMP-1, -2, and -7 secretion by cultured colon cancer cells. Recently, Zhang et al. [37] obtained similar results, i.e., that LYC decreases MMP-2, -7, and -9 in addition to N-cadherin and vimentin. They attributed those results to effects on p38 and Akt signaling pathways. LYC was also shown to affect the JAK/STAT and β-catenin pathways in melanoma cells [69,70] and the β-catenin pathway in colon cancer cells [38]. Of note, MMP-7 expression is under the control of STAT3 [71], as is the expression of MMP-1, -2, and -13 [72], as well as VEGF and pentraxin 3 [73,74]. 

Like Wnt/β-catenin, the JAK/STAT3 pathway is another major key pathway involved in CRC progression and metastasis [75]. Importantly, STAT3 was recently shown to be a new direct-binding target for LYC and NAR in the context of CRC and breast cancer, respectively [76,77]. STAT3 is currently considered one of the most promising targets to combat CRC as its over-activation is found not only in the cancer cells but also in the tumor microenvironment [60,78]. Tumor-associated macrophages and cancer-associated fibroblasts secrete IL-6 that favors via JAK/STAT3 the migration and invasion of CRC cells [60]. In addition, phosphorylated-STAT3 stimulates angiogenesis and, within exosomes, 5-FU resistance [60]. Finally, STAT3 contributes to the tumor-promoting inflammatory environment [60,78]. More than 10 clinical trials using strategies to combat STAT3 in CRC are being or have been conducted [78]. Whether HAE also could bind directly to STAT3 has not been investigated to date, at least to the best of our knowledge, but this key target in CRC makes the Amaryllidaceae alkaloids NAR, LYC, and potentially HAE as STAT3 binding agents promising for this specific cancer type. The inhibitory effects of the Amaryllidaceae alkaloids on STAT3 may thus also at least partly explain the results of our study, in particular the decrease in MMPs, VEGF, and pentraxin 3, all of which are under STAT3 transcriptional control. This is particularly true when considering the close interaction of STAT3 and NF-κB members in regulating both separately or together the expression of numerous genes (e.g., MMP-1) involved in cancer initiation, metastasis, and resistance to therapies [73,79]. Although pentraxin 3 was initially described as an oncosuppressor [80], its expression in cancers has been shown to differ depending on the cancer types [81]. For example, pentraxin 3 expression is known to increase in the initiation of inflammatory processes where it plays a role in the complement pathway [81]. In colorectal cancer, pentraxin 3 is associated with poor prognosis [82,83] but no mechanistic studies have clearly elucidated its effects. 

As mentioned above, loss of p53 function has been linked to increased NF-κB signaling, TNF sensitivity, EMT, and invasion [26]. NF-κBs are central pro-inflammatory transcription factors known to regulate the expression of numerous cytokines, including IL-1, -2, -6, -8, -12, and TNF-α, as well as MMPs and adhesion molecules such as intracellular adhesion molecule 1 (ICAM-1) and vascular cell adhesion molecule 1 (VCAM-1) leading to the initiation and development of inflammation [84]. In cancers, NF-κB is considered an oncogene involved in tumorigenesis, cancer cell proliferation, and resistance to apoptosis and is a key player in the cancer microenvironment cells as well as their crosstalk with the cancerous cells [85], particularly in CRC, where NF-κB is being considered as a potential biomarker [86].

Importantly, the literature investigating the potential anti-inflammatory effects of the Amaryllidaceae alkaloids has shown that LYC and NAR often exert their protective effects against induced-inflammatory disease models by inhibiting the NF-κB pathway, avoiding rises in TNF-α, IL-6, and IL-1β [24,29,32,34,36,87] as well as MMP-3 and MMP-13 (LYC; [20]), ICAM and VCAM (NAR; [88]) for example. We observed in the present study that NAR, LYC, and also HAE, whose anti-inflammatory effects have never been reported before to our knowledge, decrease MMP-1, -2, and -7, as well as VEGF in CRC cell supernatants (Figure 4 and Figure 5). All these proteins are associated with a poor prognosis for CRC patients [49]. MMP-1, -2, and -13 gene expression is under NF-κB control, with each MMP having one *cis*-acting enhancer in their promoter regions for NF-κB, according to Clark et al. [72], in addition to other transcription factors. In addition, VEGF might be directly and indirectly under the control of NF-κB [73], as is pentraxin 3 [89]. In the work of Zhao et al. [35], the inhibitory effects of NAR on LPS-induced neuro-inflammation were mediated by reduced NF-κB activity attributed partly to direct catalytic inhibition of its upstream activators, IKKα/β. Based on these studies, it seems possible that the effects of Amaryllidaceae observed on other inflammatory diseases might actually be also triggered in a similar manner in the context of CRC. 

The very strong effects of the three compounds on VEGF secretion should also be highlighted as anti-angiogenic therapies, and bevacizumab, in particular, is one of the treatments frequently used in combination with cytotoxic drugs, although their benefits have not been consensually approved [61]. Importantly, VEGF is recognized as a worse prognosis marker for CRC patients [50]. Decreased VEGF was previously observed in ovarian cancer cells treated with LYC (hydrochloride salt; [90]). In addition, the direct effects of NAR on endothelial cells were investigated previously by Brautigam et al. [91]. They showed inhibition of endothelial cell proliferation, migration, sprouting, and network formation mediated, at least in part, by activation of ROCK. A decrease in VEGF receptor 2 was also found and attributed to VEGF receptor 2′s short half-life associated with decreased synthesis [91].

We are currently planning in vivo work in mouse models of human colorectal cancer. Historically, most drug development research has employed xenograft models in which human colorectal cancer cells are subcutaneously injected into immune-deficient mice. This practice has several flaws that affect chemotherapeutic efficacy and reduce the successful translation of drugs from preclinical models to the clinic. Specifically, subcutaneous xenograft models ignore the contributions of the tumor microenvironment, the immune system, and microbiome to cancer progression and metastasis, as well as chemotherapeutic efficacy [92,93,94]. An ideal animal model would begin with a spontaneous colon tumor that metastasizes to the liver. Unfortunately, no such models exist as invasion and metastasis rarely occur in genetic or carcinogen-induced models of colorectal cancer [95]. Rather, we will use allograft (syngeneic) immunocompetent mouse models to account for the effects of the tissue microenvironment, immune response, and microbiome. The implantation of bioluminescent colorectal cancer cells will allow us to non-invasively monitor tumor burden over time in living animals [96]. The in vivo experiments will be initiated after the acquisition of the necessary funding.

## 5. Conclusions

The results of the present study illuminate the potential of Amaryllidaceae alkaloids to combat CRCs. In particular, the p53-independent effects on cancer cell proliferation have been discovered for the isocarbostyril, lycorane, and crinane representative compounds, a feature that is particularly important to treating advanced CRC. The results obtained with respect to the invasiveness and cytokine secretion prompted us to hypothesize that the anti-inflammatory properties observed previously with NAR and LYC in other disease models could be extended to cancer biology and may be of great value in the context of CRC therapy. All targets of Amaryllidaceae alkaloids identified to date, i.e., ribosomes, eEF1α, and recently STAT3, must be considered in the design of compounds to combat both CRC cells and the tumor microenvironment. Investigations to support key effects of Amaryllidaceae alkaloids on the CRC tumor microenvironment could open avenues to consider these compounds as multifaceted potential drug candidates and not only as general cytotoxic agents.

## Figures and Tables

**Table 1:** IC_50_ of Amaryllidaceae alkaloids determined by means of the MTT colorimetric assay after 72 h of exposure. Data are expressed in µM as the mean of six replicates of one experiment.

	Normal Cell Line (N)	Cancer Cell Lines (C)					Mean IC_50_ Cancer Cells	Ratio N/C
	CoN	LoVo	HCT-116^+/+^	HCT-116^-/-^	HT-29	DLD-1		
NAR	1.1	0.052	0.023	0.029	0.032	0.030	0.033	32
PANC	2.4	0.59	0.17	0.16	0.30	0.28	0.30	8
LYC	79	5.5	1.3	2.6	3.9	2.9	3.2	24
HAE	85	4.6	1.9	2.2	2.5	2.4	2.7	31
Cisplatin	67	8.4	4.5	17.5	22.1	14.8	13.5	5

## Data Availability

Data available upon reasonable request.
